# Visualization of the spatial positioning of the *SNRPN*, *UBE3A*, and *GABRB3* genes in the normal human nucleus by three-color 3D fluorescence in situ hybridization

**DOI:** 10.1007/s10577-012-9300-5

**Published:** 2012-07-17

**Authors:** Rie Kawamura, Hideyuki Tanabe, Takahito Wada, Shinji Saitoh, Yoshimitsu Fukushima, Keiko Wakui

**Affiliations:** 1 Department of Medical Genetics, Shinshu University School of Medicine, 3-1-1 Asahi, Matsumoto, Nagano 390-8621 Japan; 2 Department of Evolutionary Studies of Biosystems, School of Advanced Sciences, The Graduate University for Advanced Studies (Sokendai), Shonan Village, Hayama, Kanagawa 240-0193 Japan; 3 Division of Pediatric Neurology, Kanagawa Children’s Medical Center, 2-138-4, Mutsukawa, Minami-ku, Yokohama, 232-8555 Japan; 4 Department of Pediatrics and Neonatology, Nagoya City University Graduate School of Medical Sciences, 1 Kawasumi, Mizuho-cho, Mizuho-ku, Nagoya, 467-8601 Japan

**Keywords:** Genome organization, Spatial positioning, 3D-FISH, *SNRPN*, Chromatin, Epigenetic

## Abstract

The three-dimensional (3D) structure of the genome is organized non-randomly and plays a role in genomic function via epigenetic mechanisms in the eukaryotic nucleus. Here, we analyzed the spatial positioning of three target regions; the *SNRPN*, *UBE3A*, and *GABRB3* genes on human chromosome 15q11.2–q12, a representative cluster of imprinted regions, in the interphase nuclei of B lymphoblastoid cell lines, peripheral blood cells, and skin fibroblasts derived from normal individuals to look for evidence of genomic organization and function. The positions of these genes were simultaneously visualized, and all inter-gene distances were calculated for each homologous chromosome in each nucleus after three-color 3D fluorescence in situ hybridization. None of the target genes were arranged linearly in most cells analyzed, and *GABRB3* was positioned closer to *SNRPN* than *UBE3A* in a high proportion of cells in all cell types. This was in contrast to the genomic map in which *GABRB3* was positioned closer to *UBE3A* than *SNRPN*. We compared the distances from *SNRPN* to *UBE3A* (SU) and from *UBE3A* to *GABRB3* (UG) between alleles in each nucleus, 50 cells per subject. The results revealed that the gene-to-gene distance of one allele was longer than that of the other and that the SU ratio (longer/shorter SU distance between alleles) was larger than the UG ratio (longer/shorter UG distance between alleles). The UG distance was relatively stable between alleles; in contrast, the SU distance of one allele was obviously longer than the distance indicated by the genome size. The results therefore indicate that *SNRPN*, *UBE3A*, and *GABRB3* have non-linear and non-random curved spatial positioning in the normal nucleus, with differences in the SU distance between alleles possibly representing epigenetic evidence of nuclear organization and gene expression.

**Electronic supplementary material:**

The online version of this article (doi:10.1007/s10577-012-9300-5) contains supplementary material, which is available to authorized users.

## Introduction

Recent experimental and computational advances have generated spatial information about nuclear architecture. We now know that the human genome, containing some 23,000 genes and 3.2 billion base pairs of DNA, is distributed among the 22 pairs of autosomes and two sex chromosomes, all of which are packed into the current chromatin compaction model. Interphase chromosomes are generally considered to be less condensed than their mitotic counterparts. To understand the complex workings of the genome in full, it is necessary to consider its three-dimensional (3D) organization, rather than relying on linear information alone (Laster and Kosak 2010; Joffe et al. 2010). According to recent studies, higher-order chromatin organization and the spatial arrangement of genomic regions within the nucleus seem to play an important role in genome function via epigenetic mechanisms (Sproul et al. 2005; Lanctôt et al. 2007; Fraser and Bickmore 2007; Takizawa et al. 2008; Solovei et al. 2009; Ferrai et al. 2010; Egecioglu and Brickner 2011). Such findings were obtained by microscopic and, more recently, non-microscopic approaches. Microscopic techniques, such as 3D fluorescence in situ hybridization (3D-FISH) analysis, which although limited in resolution, provide spatial information such as physical distance, shape, and localization at the single-cell level (Shopland et al. 2006, Cremer and Cremer 2010; Crutchley et al. 2010). In 3D-FISH, radial positions and gene-to-gene distance are analyzed by the hybridization of probes to 3D-preserved nuclei. 3D-FISH studies have shown that individual chromosomes occupy discrete compartments called chromosome territories (CTs) that do not overlap with each other while adopting a preferential radial position within the nucleus. In many cell types, the radial organization of CTs is dependent on gene density or chromosome size. For instance, in rather spherically shaped nuclei, such as in lymphocytes, gene-dense chromosomes are located more internally while gene-poor chromosomes are located more peripherally (Croft et al. 1999; Boyle et al. 2001; Cremer et al. 2001; Tanabe et al. 2002). Bolzer et al. (2005) were the first to use 24-color 3D-FISH to simultaneously detect all chromosomes in human fibroblasts of interphase nuclei, presenting 3D maps of all CTs. Some genes change their nuclear location depending on gene activity (Lanctôt et al. 2007; Meaburn et al. 2007; Solinhac et al. 2011). For example, some genes loop out from their CT when active (Volpi et al. 2000; Williams et al. 2002; Mahy et al. 2002; Chambeyron and Bickmore 2004; Küpper et al. 2007; Ferrai et al. 2010). In this way, various genome organization phenomena have been microscopically observed.

Non-microscopic studies, such as chromosome conformation capture (3C) and 3C-based analysis, including 3C-on chip or circular 3C (4C), 3C-carbon copy (5C), chromatin interaction analysis by paired-end tag sequencing (ChIA-PET), and Hi-C, which although requiring large numbers of cells, provide spatial information of physical contact between chromatin segments at a high resolution (Dekker et al. 2002; Simonis et al. 2006; Zhao et al. 2006; Dostie et al. 2006; Fullwood et al. 2009; Lieberman-Aiden et al. 2009; Handoko et al. 2011). In particular, 3C-based methods make it possible to determine genome-wide chromatin interaction frequency. In the 3C method, the frequency of spatial contacts between genomic loci is analyzed using formaldehyde cross-linking, ligation, and locus-specific PCR (Dekker et al. 2002). Several 3C and 3C-based studies have suggested that long-range chromatin interactions are involved in the epigenetic regulation of gene expression (Simonis et al. 2007; de Wit and de Laat 2012). For instance, the higher-order chromatin conformation at some loci differs between maternal and paternal alleles, and is correlated with the formation of CCCTC-binding factor (zinc finger protein) (CTCF)-dependent parent-of-origin specific loops (Murrell et al. 2004). Long-range looping interactions between genes can occur over a genomic distance of a few kb to tens of Mb (Simonis et al. 2006; Lieberman-Aiden et al. 2009; van Steensel and Dekker 2010). Furthermore, chromatin contacts not only occur between specific short functional elements, such as enhancers and promoters, but also over larger chromosomal domains, such as intrachromosomal (*cis*), interchromosomal (*trans*), and genomic environment contacts, when active genes share a transcription factory (Crutchley et al. 2010; van Steensel and Dekker 2010). Consequently, it seems that chromatin communicates as a spatial network in interphase nuclei. Such approaches complement each other by offering new insight into genomic spatial organization and function in the nucleus (Dekker 2008; Cremer and Cremer 2010; Crutchley et al. 2010, de Wit and de Laat 2012).

Despite previous findings, information on the relationship between genomic organization and function remains limited. In an attempt to further investigate, we focused on the following three genes in imprinted loci on 15q11.2–q13: *SNRPN*, which exhibits monoallelic (paternal) expression; *UBE3A*, which exhibits tissue-specific (e.g., brain) maternal expression; and *GABRB3*, which exhibits biallelic expression. Human chromosome 15q11–q13, a region subjected to genomic imprinting, is responsible for Prader–Willi syndrome (PWS) and Angelman syndrome (AS) (Horsthemke and Wagstaff 2008). The lack of a functional paternal copy of 15q11–q13 causes PWS, while the lack of a functional maternal copy of *UBE3A* causes AS. Several groups have studied the spatial organization of 15q11–q13 using 3D image analysis. For example, Nogami et al. (2000) examined the relationship between *SNRPN* and chromosome territory in human myeloid leukemia HL60 cells. Teller et al. (2007) investigated the 3D distance between PWS/AS homologous regions in human lymphocytes, fibroblasts, and a gorilla lymphoblastoid cell line to examine the “chromosome kissing” hypothesis during the late S phase of interphase. Rauch et al. (2008) studied chromatin architecture within the PWS locus in a human lymphoblastoid cell line and fibroblast cell nuclei. They measured 3D distance between two of four probes located within 230 kb and analyzed chromatin compaction using computer simulations. However, they found no clearly detectable differences between the active and inactive PWS domains.

Although various observations have accumulated regarding the imprinted regions of PWS/AS, there are still insufficient data from a spatial viewpoint with regard to the relationship between higher-order chromatin configuration and gene activity. To the best of our knowledge, this is the first study to use three-color 3D-FISH to investigate spatial organization in the PWS/AS regions of three consecutive genomic regions––*SNRPN*, *UBE3A*, and *GABRB3*––in the nuclei of human B lymphoblastoid cell lines (LCLs), peripheral blood (PB) cells, and skin fibroblasts (FBs) derived from normal individuals.

 In this study, we measured all 3D inter-gene distances between two of three target genes on each homologous chromosome in each cell to search for new evidence of genomic organization and function. As activity of the imprinted genes differs according to parental origin, simultaneous visualization of the genes by three-color 3D-FISH at the single-cell level was the only feasible approach, regardless of advances in 3C and 3C-based analyses. Here, we report both the regularity and differences in spatial organization among the three target regions in the nucleus. Our results provide possible epigenetic evidence of a relationship between gene-to-gene distance and genome function.

## Materials and methods

### Cell materials and preparation of specimens

Epstein–Barr virus-transformed human B LCLs, mononuclear cells isolated from whole heparinized PB cells, and FBs from a healthy female individual with normal karyotype (F-LCL, F-PB, and F-FB), and LCLs and PB cells from a healthy male individual with normal karyotype (M-LCL and M-PB) were obtained for 3D-FISH analyses. Ethical approval for this project was granted by the Institutional Review Board of Shinshu University School of Medicine.

PB cells were isolated by Ficoll–Paque density gradient centrifugation, and red blood cells were removed using RBC Lysis Solution (Qiagen). PB cells were resuspended in saline at a concentration of approximately 1 × 10^7^ cells/mL.

LCLs and FBs in culture were synchronized for collection of large cell populations at G1 phase by the double-thymidine block method according to the standard procedure (Harper 2005) with minor modifications to analyze under conditions similar to PB cells at G0 phase. The releasing time was decided according to the doubling time of each cell type. LCLs were maintained in 10 % fetal bovine serum (FBS)/Roswell Park Memorial Institute 1640 (RPMI) medium at 37 °C in an atmosphere of 5 % CO_2_. Exponentially growing LCLs were blocked with excess thymidine (2 mM) for 12 h, and released for 12 h, then blocked again for 12 h, after that they were released for 15 h to synchronized G1 phase. LCLs were resuspended in 10 % FBS/RPMI at a concentration of approximately 1 × 10^7^ cells/mL. Suspended PB cells and LCLs from each subject were incubated at 37 °C for 1 h on poly-l-lysine-coated glass coverslips (24 × 60 mm). FBs were grown on coverslips with 10 % FBS/Dulbecco’s modified Eagle’s medium at 37 °C in 5 % CO_2_ and were blocked with 2 mM thymidine for 12 h, then released for 12 h, and blocked again at 12 h, after that they were released for 15–15.5 h. The percentages of the cell cycle phase fractions of G0/G1, S, and G2/M in cultured cells were analyzed using FACSCalibur and CellQuest Pro software (Becton Dickinson). More than 75 % G0/G1 cell populations of synchronized LCLs and FBs were used for 3D-FISH analyses.

All cell materials on coverslips were fixed and prepared to obtain 3D preserved cell nuclei according to the methods described previously (Cremer et al. 2001; Solovei et al. 2002) with slight modifications as follows. All coverslips with cells for 3D-FISH analysis were briefly washed with phosphate-buffered saline (PBS), fixed in 4 % paraformaldehyde (PFA) in 0.3 × PBS for 10 min, and washed again in PBS. For permeabilization, cells were treated with 0.5 % saponin and 0.5 % Triton X-100 in PBS for 20 min, washed in PBS, and after incubation in 20 % glycerol in PBS for at least 30 min, subjected to repeated freeze–thaw cycles in liquid nitrogen five times. After washing cells again in PBS, they were incubated for 10 min in 0.1 N HCl, washed in PBS, incubated in 0.002 % pepsin in 0.01 N HCl at 37 °C for 2–6 min, and washed with 0.05 M MgCl_2_ in PBS. Cells were postfixed with 1 % PFA in PBS for 10 min, washed in PBS, and then in 2× SSC for 5 min. Cells on coverslips were stored at 4 °C in 50 % formamide in 2× SSC until hybridization.

### FISH probes

For measurement of gene-to-gene 3D distance in nuclei, we focused on one of the representative clusters in a human imprinting region that includes the *SNRPN*, *UBE3A*, and *GABRB3* genes mapped on 15q11.2–q12 within the region responsible for PWS and AS. *SNRPN* is a gene with paternal-only expression, *UBE3A* is the gene responsible for AS and shows maternal > paternal tissue-specific expression, and *GABRB3* is expressed from both parental alleles (Horsthemke and Wagstaff 2008).

**Fig. 1:**
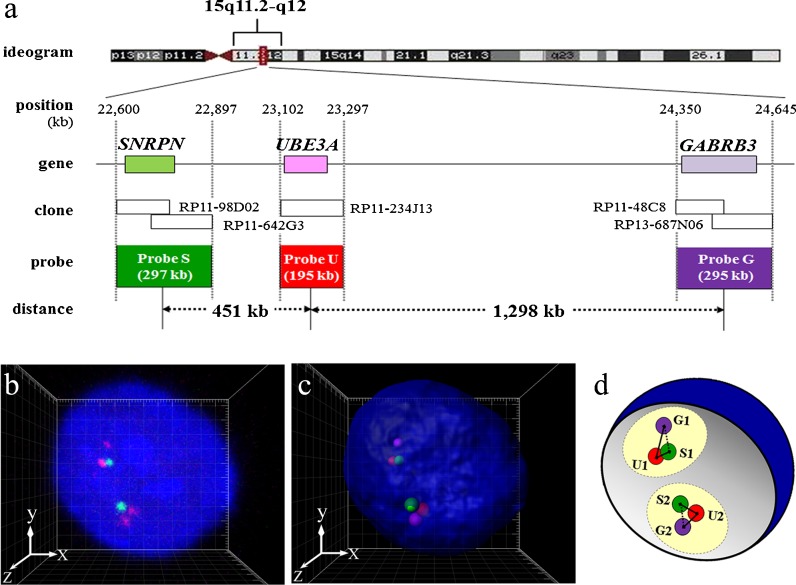
**a** Probe design for three-color 3D-FISH analysis of the target region on human chromosome 15q11.2–q12. **b**, **c** Visualization of three-color 3D-FISH on structurally preserved human LCL nuclei and an image of 3D distance measurements. FISH with probes S (*green*), U (*red*), and G (*magenta*), showing the *SNRPN*, *UBE3A*, and *GABRB3* genes, respectively. Nuclei were counterstained with DAPI (*blue*). 3D reconstruction (**c**) was carried out from the captured image (**b**) obtained with Imaris software. Each signal spot was generated using the coordinate value from the FPC of each probe (i.e., probes S (*green*), U (*red*), and G (*magenta*)). Grid space, 1 μm. **d** Scheme of the relative 3D intergenic distance measurements. *Circles* colored *light yellow* represent the assumed chromosome territories 15. SIU1 < S2U2 distance

Five human bacterial artificial chromosome (BAC) clones were selected by genome data base and purchased from BACPAC Resources at Children’s Hospital and Research Center (Oakland) as three target regions of FISH probes. The probe S region including the *SNRPN* gene (RP11-98D02 and RP11-642G3), the probe U region including *UBE3A* (RP11-234J13), and the probe G region including *GABRB3* (RP11-48C8 and RP13-687N06) (Fig. 1a). Each BAC clone DNA was cultured and extracted using the standard alkaline lysis mini-prep protocol and tested for correct chromosomal location and the absence of signals on the pericentromeric region of one chromosome 15 homolog using metaphase spreads of LCLs from a PWS patient with a deletion of 15q11.2–q13 (PWS-del) by FISH. It was confirmed that the signal of each BAC clone was absent on one chromosome 15q homolog of the metaphase from PWS-del.

According to the primary structure of the human genome, the center of probe S to the center of probe U (SU region) is physically separated by about 451 kb, and the center of probe U to the center of probe G (UG region) is about 1,298 kb; thus SU region:UG region = 0.35:1. If chromosome condensation occurs over the entire chromosome, this proportion must remain the same.

### Three-color 3D-FISH and probe detection

About 0.5 μg of DNA from each probe was used for each hybridization. Probe S, probe U, and probe G were labeled using a nick-translation kit (Abbott) with SpectrumGreen-dUTP, SpectrumOrange-dUTP (Abbott), and Cy5-dCTP (GE Healthcare), respectively, according to the manufacturer’s protocol, to measure gene-to-gene distances on each homologous chromosome 15.

3D-FISH and probe detection were performed according to protocols described elsewhere (Cremer et al. 2001; Solovei et al. 2002) with slight modifications.

Labeled probe DNAs of three target regions and Cot-1 DNA were mixed and subjected to ethanol precipitation, and then resuspended in hybridization solution (50 % formamide and 10 % dextran sulfate in 2× SSC). The probes were predenatured at 80.5 °C for 6 min and placed on ice for 1 min. Denatured probes were applied to the coverslips on which fixed cells, covered with smaller coverslips (18 × 18 mm), and sealed. The coverslip specimens were denatured at 75 °C for 5 min, and hybridization was performed in a moist chamber at 37 °C for 3–4 days. The specimens were washed in 2× SSC, 0.1× SSC at 60 °C, 4× SSC with 0.2 % Tween 20, and 4× SSC. Nuclear DNA was counterstained with 4′,6-diamidino-2-phenylindole (DAPI) and the slides were mounted in Vectashield Antifade (Vector).

### Confocal microscopic Image

Nuclei were scanned with a four-channel laser-scanning confocal microscope (Zeiss LSM5 EXCITER; Carl Zeiss MicroImaging GmbH) equipped with a Plan-Apochromat 63×/1.4 Oil DIC objective lens. For each optical section, images were collected sequentially for four fluorochromes (SpectrumGreen, SpectrumOrange, Cy5, and DAPI) using blue diode (405 nm), argon (488 nm), and helium-neon (543/633 nm) lasers, respectively.

To improve the signal-to-noise ratio, each sectional image obtained was an average of two successive scans. The focus *z*-step between sections was 0.364 μm. Stacks of 12-bit grayscale two-dimensional images were obtained with 512 × 140–320 pixels in each channel.

Confocal image stacks were processed with the microscope operating software (ZEN; Carl Zeiss MicroImaging GmbH) and saved as LSM files. More than 50 nuclear images were captured from each cell material. Nuclei from cultured cells with singlet-singlet signals were adopted for calculation as in G1 phase of the cell cycle but with doublet-doublet or singlet-doublet signals for each probe, which were suspected to be in S or G2 phase, were not selected for capture.

### Quantitative 3D evaluation

We specified the 3D coordinates of three target regions at a time in each cell and calculated the actual measured value between two of the three regions, and then determined the spatial organization among these regions in the nucleus. Various 3D measurement data, such as the coordinate value of the fluorescence peak center (FPC) of each signal/nucleus volume/sphericity/ellipsoid axis length *x*, *y*, and *z*, were obtained using scientific 3D and 4D image processing and analysis software (Imaris, Imaris MeasurementPro, and ImarisCell; Bitplane).

Nuclei with sphericity of <0.5, suspected to be unable to maintain initially ordered 3D structures of the cells, were excluded from the calculation as the deformed nuclear shape leads to distortion of gene topology. Finally, 50 nuclei of each cell material were analyzed.

We measured the relative 3D gene-to-gene distance of three target regions, *SNRPN* (S), *UBE3A* (U), and *GABRB3* (G) genes at 15q11.2–q12 on each homologous chromosome 15 within the interphase nuclei, beginning with the 3D coordinate value of FPC of six fluorescent signals of the probes determined while checking 3D images of each nucleus simultaneously. We defined as “allele 1” on one of the homologous chromosomes 15 that had a shorter probe S-to-U distance (SU distance) than the other homologous chromosomes 15, and the FPC of probes S/U/G were defined as S1/U1/G1 on allele 1 in each nucleus. The FPC of probes S/U/G were defined as S2/U2/G2 on allele 2, which had a longer SU distance in each nucleus. Diagram of 3D distance measurements is shown in Fig. 1d. The shortest physical distances between two of the three probes—SU distance, UG distance, and SG distance—on each homologous chromosome 15 were calculated from the *x*, *y*, and *z* coordinates of the FPC of signals using the following equation and the spreadsheet application Excel (Microsoft Corporation).

${\delta_{{ij}}} = \sqrt {{{{\left( {{x_i} - {x_j}} \right)}^2} + {{\left( {{y_i} - {y_i}} \right)}^2} + {{\left( {{z_i} - {z_i}} \right)}^2}}}$ *any two loci *i* and *j*

We also calculated angle U, which was defined as an internal angle formed by SU and UG sides, from the measurement data of distances SU, UG, and SG using the second cosine theorem with the following equation and the spreadsheet application Excel (Microsoft Corporation).

$\theta = {\cos^{{ - 1}}}\frac{{{b^2} + {c^2} - {a^2}}}{{2 \times b \times c}}$ *θ: angle U, side *b*/*c*/*a*: distance SU/UG/SG

### Statistical analysis

Fifty nuclei of each cell material were examined. We performed exploratory data analysis to find patterns in our results. Normality was assessed with the Shapiro–Wilk test. The distribution of measurements in a proportion of samples was not normal. Therefore, all measurements were analyzed using the nonparametric Mann–Whitney test between different cell types within the same individuals and between the same cell types among individuals. All statistical tests were two-sided, and *P* < 0.05 was considered to indicate statistical significance. For multiple comparisons, significance levels were modified according to Bonferroni’s correction (*α*). All statistical analyses were performed with SPSS software version 18.0 (IBM).

## Results

**Fig. 2:**
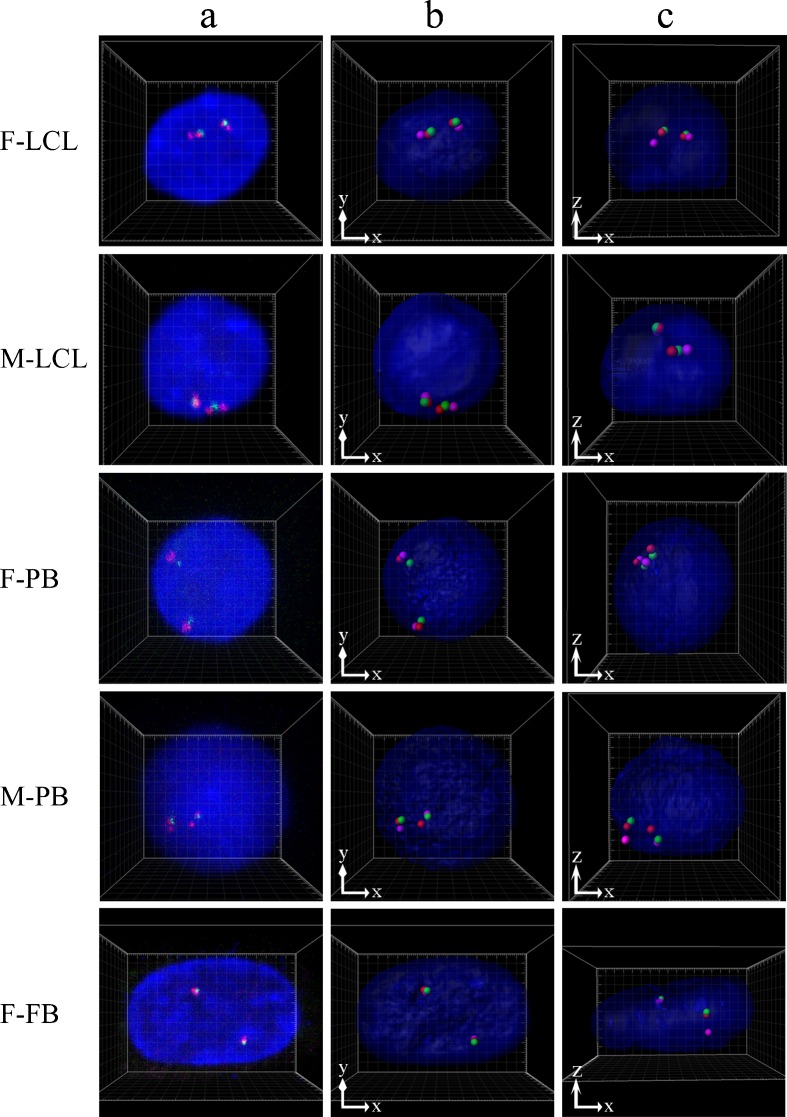
Examples of three-color 3D-FISH results of projections and 3D reconstructions in typical nuclei from each subject. *Green*/*red*/*magenta* signals: probes S/U/G. 3D reconstructions in the *xy* (**b**) and *xz* direction (**c**) were obtained from the captured image (**a**) generated by Imaris software. Grid space, 1 μm

Three-color 3D-FISH was performed to measure gene-to-gene distance on 15q11.2–q12, on each homologous part of chromosome 15, within each interphase nucleus from the three different cell types examined (Figs. 1 and 2). The 3D gene-to-gene distance, angle U from 100 alleles in 50 nuclei, and the radius, volume, and sphericity of 50 nuclei in each subject are summarized in Table 1. Values were corrected according to the average *x*-, *y*- and *z*-axis radius to enable comparisons of gene-to-gene distance between different subjects.

**Table 1:** 3D distances of SU/UG/SG and angle U in the nucleus calculated using the 3D coordinate values of the FPC of probe signals and the radius, volume, and sphericity of the nucleus

Subjects	Measured value median (IQR; μm)			Measured value median (IQR)			
	Corrected value median^a^ (IQR; %)						
	SU distance	UG distance	SG distance	Angle U^b^ (degree)	Radius^c^ (μm)	Volume (μm^3^)	Sphericity
F-LCL	0.255 (0.170–0.366)	0.645 (0.436–0.864)	0.564 (0.352–0.771)	56 (34–73)	4.2 (4.0–4.3)	290 (264–318)	0.93 (0.91–0.94)
	6.2 (4.0–8.6)	15.7 (10.4–20.6)	13.5 (8.2–18.3)				
M-LCL	0.345 (0.248–0.523)	0.803 (0.571–1.000)	0.722 (0.504–0.898)	59 (41–86)	5.4 (5.2–5.5)	605 (531–637)	0.83 (0.77–0.88)
	6.6 (4.8–10.0)	15.2 (11.2–19.1)	13.7 (9.5–17.6)				
F-PB	0.439 (0.268–0.719)	0.945 (0.726–1.285)	0.895 (0.677––1.243)	59 (37–85)	5.0 (4.7–5.3)	515 (430–597)	0.93 (0.87–0.94)
	8.3 (5.4–13.8)	18.7 (14.2–25.8)	17.7 (13.3–25.0)				
M-PB	0.569 (0.326–1.068)	1.046 (0.759–1.434)	0.964 (0.68–1.324)	63 (34–88)	5.2 (4.5–5.7)	560 (372–710)	0.91 (0.88–0.94)
	10.8 (6.7–18.6)	21.1 (15.0–26.6)	19.4 (13.7–26.0)				
F-FB	0.423 (0.271–0.556)	0.806 (0.593–1.057)	0.674 (0.470–0.900)	53 (31–84)	6.1 (5.6–7.4)	683 (565–1152)	0.70 (0.65–0.75)
	6.6 (4.3–9.0)	12 (9.5–15.9)	9.9 (7.5–14.3)				

*n* = 100 alleles, 50 nuclei from each subject

*IQR* interquartile range

^a^Median of corrected value by the radius (relative radius (in percent)). Measured value/radius × 100

^b^Internal angle formed by SU and UG sides

^c^Average of *x*-, *y*-, and *z*-axis radius

### Gene-to-gene distance of the target regions and spatial positioning

A set of three signals for probes S, U, and G were readily distinguished on each allele in all cells. The interquartile range (IQR) and medians of the SU/UG/SG distance are shown in Table 1. Overall, the SG distance was shorter than the value obtained by summing the SU and UG distance.

**Fig. 3:**
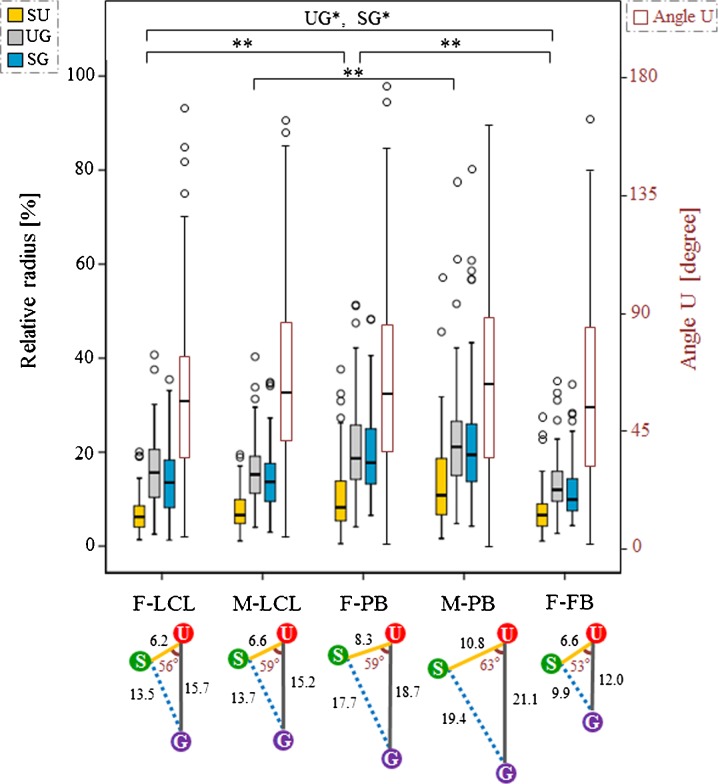
Gene-to-gene distance of SU/UG/SG and angle U for each subject. The *colored box* and *whisker plots* show the distributions of SU, UG, and SG gene distance (corrected value, relative radius), and the *red box* and *whisker plots* show the distributions of angle U. Angle U is defined as the internal angle formed by the SU and UG sides. The *left* axis in the graph shows the relative radius and the *right* axis the angle U. The *box plot* summarizes data obtained using the median, upper, and lower quartiles, as well as the range. *Boxes* represent the 25th to 75th percentiles (IQR). The *solid line* within the boxes indicates the median. *Lower* and *upper whiskers* show the 10th and 90th percentiles, respectively, of the distribution. *Open circles* indicate outliers. For the SU/UG/SG distance and the angle U, *P* values were obtained using the Mann–Whitney test between different cell types of the same individuals (F-LCL, F-PB, and F-FB; M-LCL and M-PB) and between identical cell types from different individuals (F-LCL and M-LCL; F-PB and M-PB). A *P* value < 0.008 was considered statistically significant after correcting for multiple comparisons (Bonferroni’s correction, *α* = 0.05/6 = 0.008; *P < 0.008; **P < 0.001; *n* = 100 alleles, 50 nuclei) The *bottom schema* presents the summarized configuration of *SNRPN*, *UBE3A*, and *GABRB3* genes in the nucleus for each subject, cited according to the corrected median value of the SU/UG/SG distance and the angle U (Table 1)

As the volume of the nucleus varied between subjects, and since gene-to-gene distance is thought to be influenced by nuclear volume (as shown in Table 1), comparisons of distance were made after normalizing the average nuclear radius of the *x*-, *y*-, and *z*-axis in each subject (Fig. 3). The SU/UG/SG distance were significantly different between LCLs, PB cells, and FBs of the same individual (*P* < 0.0005, UG and SG distances between F-LCLs and F-FBs; *P* = 0.004, *P* = 0.007, respectively; Bonferroni’s correction, *α* < 0.008), except for the SU distance between F-LCLs and F-FBs. There was no significant difference between identical cell types from different individuals for LCLs and PB cells (F-LCLs and M-LCLs; F-PB and M-PB cells) (Fig. 3).

Angle U, defined as the internal angle formed by the SU and UG sides, also varied in size, with the median angle being approximately 60° in all subjects (Table 1; Fig. 3). There was no significant difference between different cell types of the same individual (F-LCLs, F-PB cells, and F-FBs; M-LCLs and M-PB cells), and between identical cell types from different individuals (F-LCLs and M-LCLs; F-PB and M-PB cells).

### Distance ratio between alleles and between regions

**Fig. 4:**
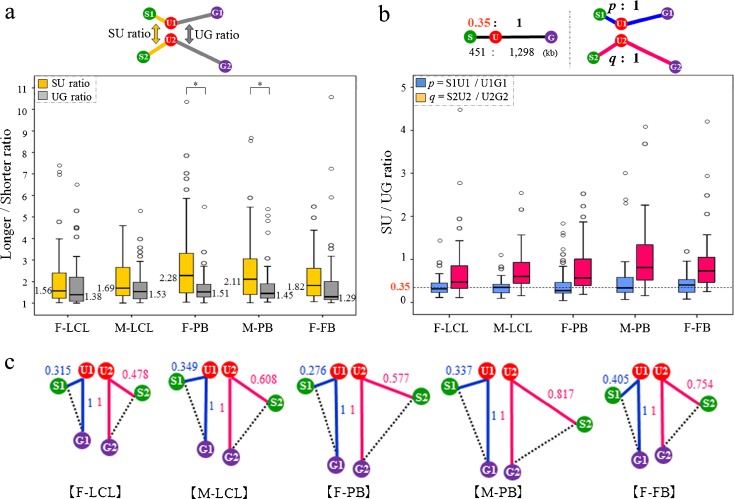
Distance ratios between alleles and between regions. **a** SU and UG distance ratios between alleles in each cell for each subject. *Box* and *whisker* plots show the distributions of the distance ratio for each subject. Distance ratios were calculated as follows: SU ratio (*yellow lines* in the top diagram) = longer SU/shorter SU distance; UG ratio (*gray lines* in the top diagram) = longer UG/shorter UG distance. For both the SU and UG ratios, *P* values were obtained using the Mann–Whitney test within each subject and between subjects (F-LCL, F-PB, and F-FB; M-LCL and M-PB; F-LCL and M-LCL; and F-PB and M-PB). A *P* value < 0.0045 was considered statistically significant after correcting for multiple comparisons (Bonferroni’s correction, *α* = 0.05/11 = 0.0045; *P < 0.004; *n* = 50 nuclei). **b** SU/UG distance ratios of each allele for each subject. In the primary sequence of the human genome, SU:UG = 451 kb:1,298 kb = 0.35:1 (*upper left* *diagram*). Ratio of the distance of S1U1when the distance of U1G1 was set to 1 (*blue lines* in the upper right diagram): *p* = S1U1/U1G1 (*blue box* and *whisker plots* in the graph). Ratio of the distance of S2U2 when the distance of U2G2 was set to 1 (*red lines* in the upper right diagram): *q* = S2U2/U2G2 (*red box* and *whisker plots* in the graph). The *dashed line* in the plot shows 0.35. For *p* and *q*, *P* values were obtained using the Mann–Whitney test between different cell types of the same individual (F-LCL, F-PB, and F-FB; M-LCL and M-PB) and between identical cell types from different individuals (F-LCL and M-LCL; F-PB and M-PB). A *P* value < 0.0045 was considered statistically significant after correcting for multiple comparisons (Bonferroni’s correction, *α* = 0.05/6 = 0.008); no significant difference was observed (*n* = 50 nuclei). **c** Configuration of *SNRPN*, *UBE3A*, and *GABRB3* genes on homologous chromosomes in the nucleus for each subject drawn based on Table 2 and (**b**). Decimal fractions in *blue* indicate the median distance ratio of *p* as shown in (**b**). Likewise, decimal fractions in red denote the median distance ratio of *q*. The blue value “1” and red value “1” are not equal distances

We analyzed the 3D intergenic distance of three target genes between each homologous part of chromosome 15 for each allele in all subjects (Fig. 1d) to determine differences between alleles of each target region (e.g., S1U1 vs. S2U2) (Fig. 4a) and between adjacent parts of the same chromosome (e.g., S1U1 vs. U1G1) (Fig. 4b) within 3D nuclei. The median distance of S1U1, S2U2, U1G1, and U2G2 is shown in Table 2.

**Table 2:** Relative 3D distances of SU/UG/SG for each allele in the nucleus

Subjects	S1U1 distance (μm)	U1G1 distance (μm)	S1G1 distance (μm)	S2U2 distance (μm)	U2G2 distance (μm)	S2G2 distance (μm)
	Median (IQR)	Median (IQR)	Median (IQR)	Median (IQR)	Median (IQR)	Median (IQR)
F-LCL	0.194 (0.124–0.273)	0.615 (0.429–0.765)	0.555 (0.360–0.740)	0.353 (0.251–0.448)	0.711 (0.440–0.933)	0.582 (0.350–0.893)
M-LCL	0.270 (0.166–0.334)	0.770 (0.597–0.936)	0.750 (0.638–0.878)	0.510 (0.356–0.601)	0.834 (0.547–1.038)	0.681 (0.433–0.901)
F-PB	0.273 (0.189–0.431)	0.908 (0.689–1.206)	0.856 (0.673–1.123)	0.652 (0.442–0.994)	0.987 (0.732–1.541)	0.968 (0.680–1.320)
M-PB	0.365 (0.228–0.594)	1.081 (0.756–1.424)	1.030 (0.754–1.319)	0.934 (0.549–1.187)	1.020 (0.781–1.437)	0.910 (0.581–1.331)
F-FB	0.271 (0.206–0.432)	0.852 (0.591–1.086)	0.692 (0.499–1.014)	0.531 (0.409–0.725)	0.803 (0.604–0.988)	0.657 (0.451–0.818)

*n* = 50 nuclei from each subject

*IQR* interquartile range

The distance ratio was defined as:

- SU ratio = longer SU/shorter SU distance
- UG ratio = longer UG/shorter UG distance

In all subjects, the median SU ratio was higher than the median UG ratio. The differences between the SU and UG ratios were significant in F-PBs and M-PB cells (*P* = 0.0004 and *P* = 0.0037, respectively; Bonferroni’s correction, *α* < 0.0045). There was no significant difference in the SU and UG ratio between different cell types of the same individual (F-LCLs, F-PB cells, and F-FBs; M-LCLs and M-PB cells) and between identical cell types from different individuals (F-LCLs and M-LCLs; F-PB and M-PB cells) (Fig. 4a).

According to the genomic coordinates, SU is 451 kb and UG is 1,298 kb, therefore SU:UG = 0.35:1. The median distance ratios were: S1U1/U1G1 = 0.32, S2U2/U2G2 = 0.48 in F-LCLs; S1U1/U1G = 0.35, S2U2/U2G2 = 0.61 in M-LCLs; S1U1/U1G = 0.28, S2U2/U2G2 = 0.58 in F-PB cells; S1U1/U1G = 0.34, S2U2/U2G2 = 0.82 in M-PB cells; and S1U1/U1G1 = 0.41, S2U2/U2G2 = 0.75 in F-FBs (Fig. 4b, c). There was no significant difference in allele 1 between different cell types of the same individual (F-LCLs, F-PB cells, and F-FBs; M-LCLs and M-PB cells) and between identical cell types from different individuals (F-LCLs and M-LCLs; F-PB and M-PB cells). Similarly, there was no significant difference in allele 2 (Fig. 4b).

## Discussion

The 3D structure of the genome is organized non-randomly and plays a role in genome function via epigenetic mechanisms in the human nucleus. However, the genome is far more complex than can be explained by linear information alone. The present study was therefore performed to investigate how consecutive genes including imprinting genes are arranged spatially in human interphase nuclei with the aim of acquiring knowledge of genomic organization and function. We focused on *SNRPN*, showing paternal expression only, and contiguous *UBE3A* and *GABRB3* genes and examined whether specific higher-order chromatin organization could be observed microscopically using three-color 3D-FISH analysis in normal LCLs, PB cells, and FBs, all of which are used frequently for research (Figs. 1 and 2). Next, we evaluated regularity and differences in their spatial positioning (Figs. 3 and 4).

### Gene-to-gene distances and spatial positioning of the target regions

We found that the *SNRPN*, *UBE3A*, and *GABRB3* genes had non-linear and non-random curved spatial organization in the human nucleus (Fig. 3). Microscopic observations indicated that a distance of about 500 kb was measurable for comparison between homologous parts on the chromatin. Rauch et al. (2008) found no clearly detectable differences between the active and inactive PWS domains, as measured by 3D distance between two of four probes located within 230 kb. Our results indicated that *GABRB3* tended to be located closer to *SNRPN* than *UBE3A*, in contrast to the genomic map. Moreover, the median values of the internal angle U were calculated as 53–63° (Fig. 3). 4C analysis of human neuronal cells revealed that a PWS-imprinting center forms chromatin loops that contain key neurodevelopmental genes, including *GABRB3* (Yasui et al. 2011). Our results showing non-linear and non-random curved spatial organization of this region may support these findings.

### Distance ratios between alleles and regions

We also found that gene-to-gene distance was not similar in size between alleles and regions (Fig. 4). The UG distance tended to be stable between alleles compared with the SU distance, even though the physical distance of UG is longer than that of SU according to the primary structure (Table 2). Figure 4a shows the differences in the SU and UG distance ratio between alleles; the SU ratio was larger than the UG ratio in all subjects. Comparison of the distance ratios between regions on the same allele revealed that the ratios of S2U2/U2G2 were >0.35, although those of S1U1/U1G1 were around 0.35 in all subjects (Fig. 4b). These results suggest that the S2U2 region may loosen more than the other region. It is generally believed that actively transcribed genes or genes poised for transcription are present in decondensed “open” chromatin configurations, while permanently silent genes are located within compact “closed” chromatin (Cremer et al. 2006). Regarding the PWS/AS region, Ohta et al. (1999) demonstrated that *SNRPN* chromatin is found in an open configuration exclusively on the paternal-derived allele. Thus, to summarize, the above results suggest that the degree of condensation seems to differ between homologous regions and adjacent regions of SU and UG.

### Cell type specificity

The findings revealed that the spatial organization of the three target regions had a similar basic distribution in each of the three cell types examined. There were, however, subtle variations in gene-to-gene distance, which were dependent on cell type, even when from the same individual (Figs. 3 and 4). The *SNRPN* gene showed the same methylation pattern in a variety of tissues including LCLs, PB cells, and FBs (Glenn et al. 1996; Birney et al. 2010). Differences between cell types were related to the fact that PB cells and LCLs are in suspension, while FBs are adherent cells. The differences between PB cells and cultured cells were thought to be have been influenced by the cell cycle since the PB cells were all in the G0 phase while the LCLs and FBs included G1, S, G2, and M phase cells, although the cultured cells were synchronized so the majority of the cell population was in the G1 phase. In addition, PB cells are composed of several kinds of mononuclear cells, in particular, T lymphocytes, B lymphocytes, and monocytes.

In this analysis, we examined 50 cells in each subject, and the obtained values of inter-gene distance displayed large variability among not only cycling cultured cells but also PB cells. Each fixed cell nucleus evaluated in the 3D-FISH analysis seemed to represent a snapshot in time of the higher-order structure and dynamics of chromatin (Teller et al. 2007; Cremer and Cremer 2010). Some investigations have shown that the movement of chromosomes and gene loci increases during early G1 (Walter et al. 2003) while other observations focusing on short-range chromatin motion suggest that local diffusional motion of chromatin is important in gene regulation (Soutoglou and Misteli. 2007). The probabilistic positioning of chromosomes can therefore show relatively large variation when single cells are compared. Furthermore, the 3D distance between genes in the nucleus is potentially influenced by chromatin compaction. Our results from the PB cells suggest that there is a range of chromatin compaction in the nucleus. Nishino et al. (2012) reported that human mitotic chromosomes consist predominantly of irregularly arranged nucleosome fibers, which they suggested exist in a similar state in the majority of active interphase nuclei. Our results will therefore help clarify chromatin structure in future studies.

3D-FISH using the three- or more-color approach is a powerful experimental tool for simultaneously visualizing the spatial positioning of multiple regions and comparing alleles in individual cells. However, it is also necessary to take into consideration the possible effects of the complicated process used to fix the cells and maintain their 3D structure for FISH analysis. It is difficult to preserve perfectly the 3D structure of nuclei from cells in suspension culture. Indeed, in this study, the volume of some nuclei could not be reproduced (data not shown).

In conclusion, the results of this study suggest that the *SNRPN*, *UBE3A*, and *GABRB3* loci have non-linear and non-random curved spatial organization in the nuclei of normal human cells. A distance of about 500 kb was measured microscopically for comparisons between homologous parts of chromatin within the nucleus. In addition, the differences in SU distance between alleles and between regions on each chromosome 15 seem to represent new epigenetic evidence of nuclear organization and gene expression. Confirmation of the relationship between activity and the 3D distance of imprinted genes in the nucleus now remains in future studies.

If the epigenetic hypothesis is confirmed whereby intergenic distance is shown to vary depending on gene activity, it could lead to further research on the development of new diagnostic techniques for patients in whom mutations cannot be identified. This would be a breakthrough in our understanding of the pathological processes of certain diseases with unknown causes, as well as adding to basic research on chromatin structure, of which much remains unknown.

## Electronic supplementary material

Below is the link to the electronic supplementary material.

Fig. S1 Metaphase FISH result by four kinds of probes mapped on chromosomes 15 using LCLs from a patient of Prader–Willi syndrome with a deletion of 15q11.2–q13 (PWS-del). The probes S (*green*), U (*red*), and G (*magenta*), indicated by *arrows*, were the same probes using for 3D-FISH in this study (Fig. 1). The probe 15qter (RP11-89K11) (*blue*), indicated by *arrowheads*, was labeled by SpectrumAqua-dUTP (Abbott) as a control probe for chromosome 15, and were hybridized with the probes S, U, and G on the metaphase spreads of PWS-del. It was confirmed that the probes S, U and G were mapped on correct loci, as the signals of these probes were absent on one of the chromosomes 15 of PWS-del. **a** Merged image with all 4 probes; S (*green*), U (*red*), G (*magenta*), and 15qter (*blue*). Selected image with the probes S (*green*) and 15qter (*blue*) (**b**), U (*red*) and 15qter (*blue*) (**c**), and G (*magenta*) and 15qter (*blue*) (**d**) (TIFF 28436 kb) (JPEG 242 kb)

High-resolution image file (TIFF 28436 kb)

Fig. S2 Gene-to-gene relative distances of SU/UG/SG using actual measurement data of each subject for the comparison to the Fig.3 in the text. *Box* and *whisker plots* show the distributions of SU, UG, and SG gene distances (actual measured distances) from 100 alleles in 50 nuclei for each subject. The *box plots* summarize data using the median, upper, and lower quartiles, and the range. Lower and upper whiskers show the 10th and 90th percentiles, respectively, of the distribution. The boxes represent the 25th to 75th percentiles (IQR). The *solid line in the boxes* indicates the median. Outliers are shown as *open circles*. (PPTX 84 kb)

## Abbreviations

3C: Chromosome conformation capture
3D: Three dimensional
4C: 3C-on chip or circular 3C
AS: Angelman syndrome
BAC: Bacterial artificial chromosome
CT: Chromosome territory
FBs: Skin fibroblasts
FISH: Fluorescence in situ hybridization
FPC: Fluorescence peak center
*GABRB3*: Gamma-aminobutyric acid (GABA) A receptor, beta 3
IQR: Interquartile range
LCLs: B lymphoblastoid cell lines
PB: Peripheral blood
PWS: Prader–Willi syndrome
*SNRPN*: Small nuclear ribonucleoprotein polypeptide N
*UBE3A*: Ubiquitin-protein ligase E3A
