# Supplementary figures and images for: Visualization of the spatial positioning of the SNRPN, UBE3A, and GABRB3 genes in the normal human nucleus by three-color 3D fluorescence in situ hybridization
Source: Chromosome Res. 2012 Jul 17;20(6):659–72. doi: 10.1007/s10577-012-9300-5 (PMC3481056; doi:10.1007/s10577-012-9300-5)

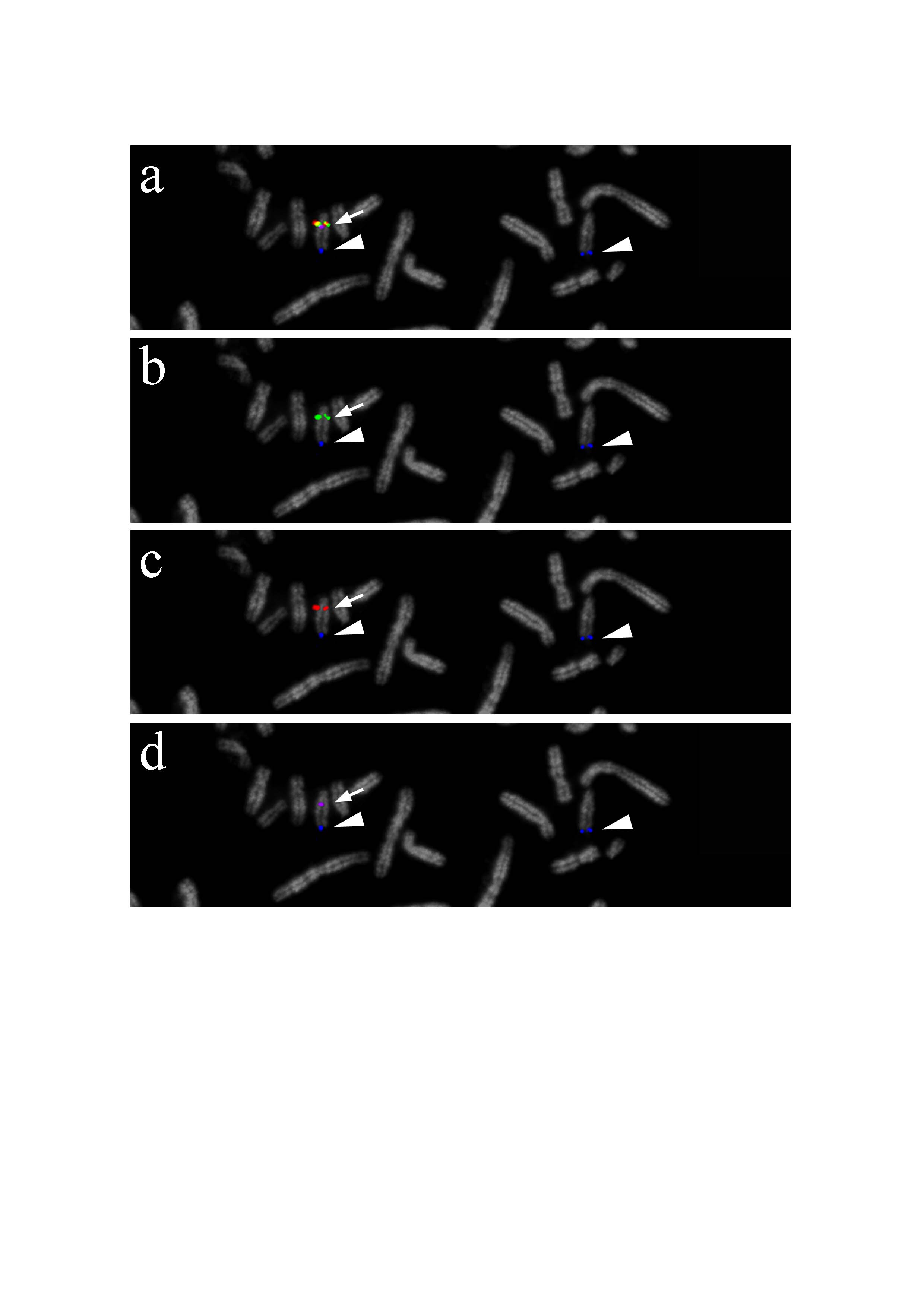

Supplement: Supplementary file 1 — Metaphase FISH result by four kinds of probes mapped on chromosomes 15 using LCLs from a patient of Prader–Willi syndrome with a deletion of 15q11.2–q13 (PWS-del). The probes S (green), U (red), and G (magenta), indicated by arrows, were the same probes using for 3D-FISH in this study (Fig. 1). The probe 15qter (RP11-89K11) (blue), indicated by arrowheads, was labeled by SpectrumAqua-dUTP (Abbott) as a control probe for chromosome 15, and were hybridized with the probes S, U, and G on the metaphase spreads of PWS-del. It was confirmed that the probes S, U and G were mapped on correct loci, as the signals of these probes were absent on one of the chromosomes 15 of PWS-del. a Merged image with all 4 probes; S (green), U (red), G (magenta), and 15qter (blue). Selected image with the probes S (green) and 15qter (blue) (b), U (red) and 15qter (blue) (c), and G (magenta) and 15qter (blue) (d) (TIFF 28436 kb) (JPEG 242 kb) [file 10577_2012_9300_Fig5_ESM.jpg]

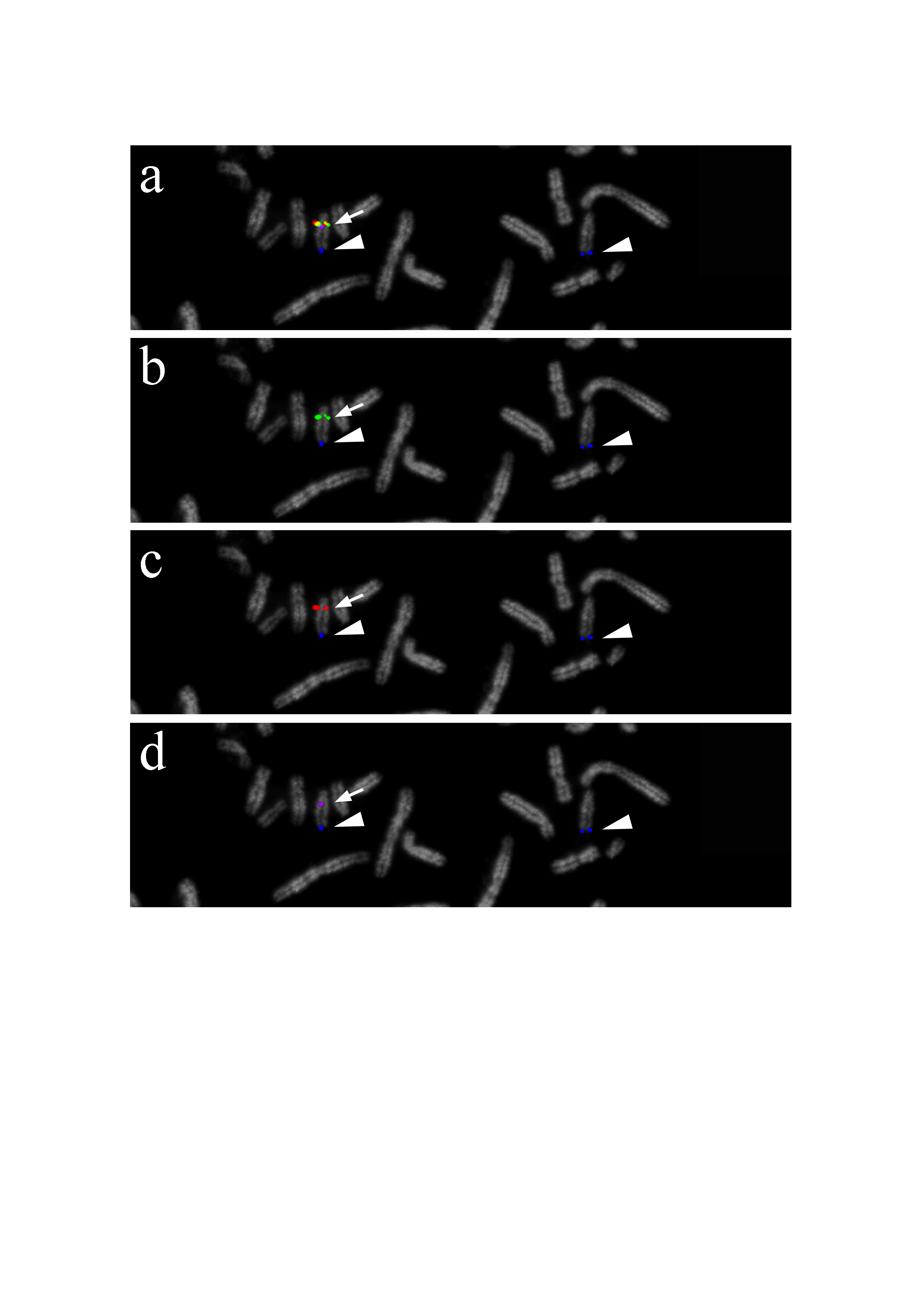

Supplement: Supplementary file 2 — High-resolution image file (TIFF 28436 kb) [file 10577_2012_9300_MOESM1_ESM.tif]

## Slide 1
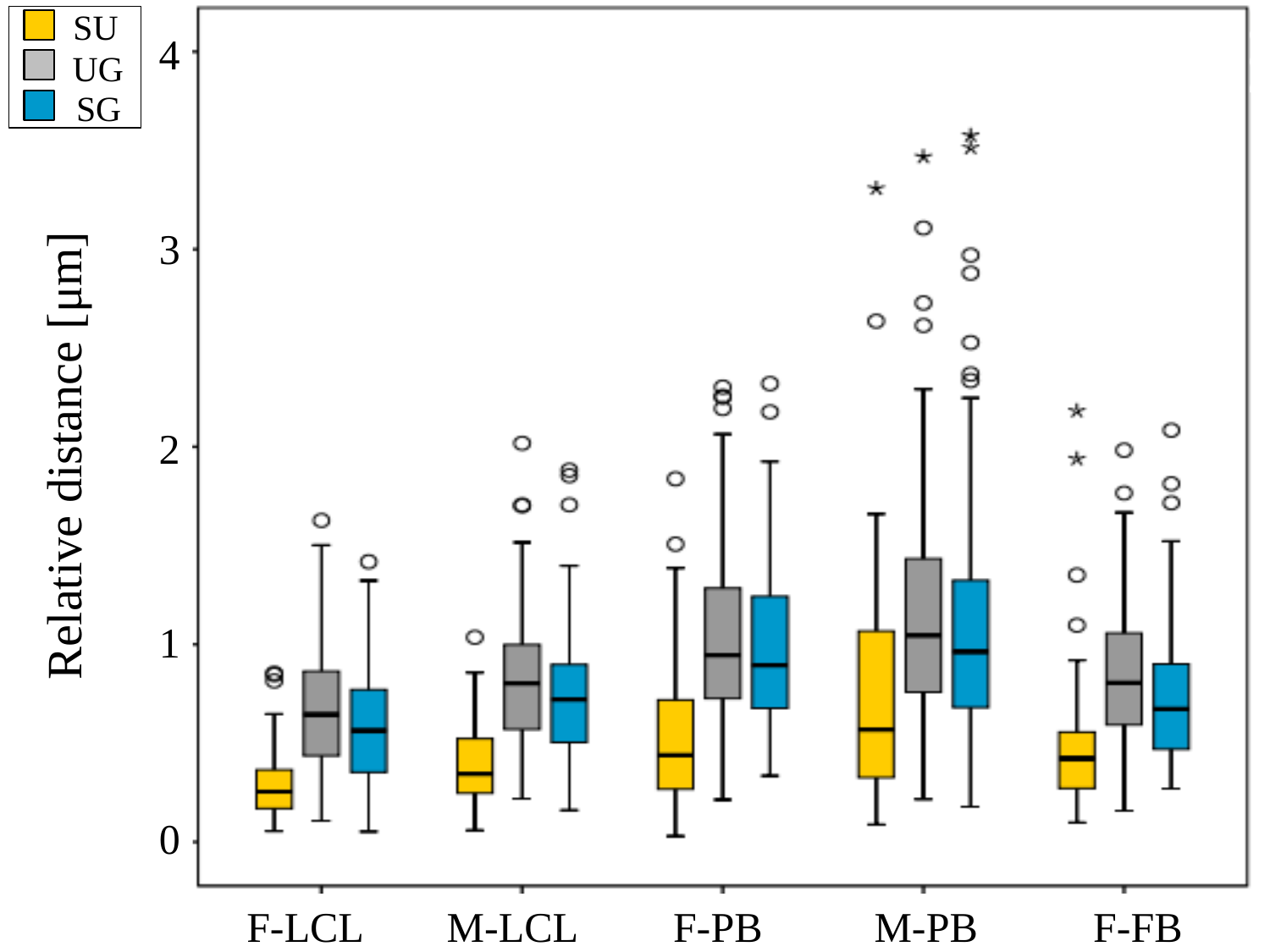

SU
UG
SG
4
3
2
1
0
Relative distance [μm]
F-FB
F-LCL
M-LCL
F-PB
M-PB

Supplement: Supplementary file 3 — Gene-to-gene relative distances of SU/UG/SG using actual measurement data of each subject for the comparison to the Fig.3 in the text. Box and whisker plots show the distributions of SU, UG, and SG gene distances (actual measured distances) from 100 alleles in 50 nuclei for each subject. The box plots summarize data using the median, upper, and lower quartiles, and the range. Lower and upper whiskers show the 10th and 90th percentiles, respectively, of the distribution. The boxes represent the 25th to 75th percentiles (IQR). The solid line in the boxes indicates the median. Outliers are shown as open circles. (PPTX 84 kb) [file 10577_2012_9300_MOESM2_ESM.pptx]
